# Assessment of hypertension control and factors associated with the control among hypertensive patients attending at Zewditu Memorial Hospital: a cross sectional study

**DOI:** 10.1186/s13104-019-4173-8

**Published:** 2019-03-18

**Authors:** Boressa Adugna Horsa, Yewondwossen Tadesse, Ephrem Engidawork

**Affiliations:** 1 Department of Clinical Pharmacy, School of Pharmacy, College of Medicine and Health Sciences, University of Gondar, P.O. BOX: 196, Gondar, Ethiopia; 2 Department of Internal Medicine, School of Medicine, College of Health Sciences, Addis Ababa University, Addis Ababa, Ethiopia; 3 Department of Pharmacology and Clinical Pharmacy, School of Pharmacy, College of Health Sciences, Addis Ababa University, Addis Ababa, Ethiopia

**Keywords:** Hypertension, Antihypertensive treatment, Blood pressure control, Ethiopia

## Abstract

**Objective:**

This study was conducted to assess hypertension control and factors associated with it among hypertensive patients on treatment at Zewditu Memorial Hospital.

**Results:**

A total of 225 patients were included in the study, of which 55.6% of patients were females. The mean age of the patients was 55.2 years and half of them had a family history of hypertension. About 29% of patients had comorbidities. Angiotensin-converting enzyme inhibitors (ACEIs), calcium channel blockers (CCBs) and beta-blockers (BBs) were the most frequently prescribed medications. Majority of (83.1%) the patients received combination therapy. The most frequent two and three drugs class combination were ACEI + BB and ACEIs + CCB + BB, respectively. Drug treatment was modified for 22.2% of patients and blood pressure (BP) was controlled in 26.2% of patients. Older age was associated with good BP control (AOR 2.58, CI 1.27–5.24), while treatment modification was associated with poor BP control (AOR 0.21, CI 0.07–0.65). The findings indicate that BP control was low and factors like middle age and treatment modification contributed to the low BP control. It is recommended that the physicians should be adherent to current guidelines regarding the selection of appropriate antihypertensive medications so as to achieve target BP goals.

**Electronic supplementary material:**

The online version of this article (10.1186/s13104-019-4173-8) contains supplementary material, which is available to authorized users.

## Introduction

Hypertension is one of the top risk factors for global mortality and is projected to have caused 9.4 million deaths and accounts for 7% of the worldwide disease burden in 2010-as measured in disability-adjusted life years [1]. Hypertension is a main cardiovascular risk factor. If left uncontrolled, high blood pressure (BP) causes myocardial infarction, heart failure, stroke, renal failure, dementia, and blindness, causing human suffering and imposing huge financial and service problems on health care systems [2].

In Low- and middle-income countries, 47% of mortality secondary to cardiovascular diseases (CVDs) and 44% of CVDs burden are attributable to high BP [3]. As per the Federal Ministry of Health of Ethiopia reports, hypertension accounted for 3% of all deaths in 2005/06, making it the 6th top cause of death in the country [4].

Even though antihypertensive treatments clearly lessens the risk of renal diseases and cardiovascular, large segments of the hypertensive patients are either untreated or inadequately treated [5]. Failure to provide lifestyle modifications, optimum antihypertensive drug doses, or correct drug combinations may result in poor BP control [6].

In Ethiopia, the prevalence of hypertension is high; however, hypertensive patients’ awareness, therapy, and control of BP rates were very low [7–11]. Thus, treatment and prevention are urgently needed. As a result, effective control of hypertension has become a priority for the Federal Ministry of Health [12]. To help with these efforts, this study attempted to assess gaps, if any, in the management of hypertension at Zewditu Memorial Hospital.

The main aim of this study was to assess hypertension control and factors associated with it among hypertensive patients on treatment at Zewditu Memorial Hospital.

## Main text

### Methods

#### Study design and study setting

A descriptive cross-sectional study was conducted at Zewditu Memorial Hospital, located in the center of Addis Ababa from June 1, 2014 to August 30, 2014.

#### Source and study population

All hypertension patients on follow-up and attending Zewditu Memorial Hospital were used as a source population. Those patients who got treatment formed the study population.

#### Inclusion and exclusion criteria

All adult hypertensive patients (> 18 years) treated at the hospital willing to participate and were on antihypertensive drug treatment for at least 6 months were included. Hypertensive patients whose chart did not have all the required information were excluded from the study.

#### Sample size and sampling technique

The sample size for the study was determined using single proportion population sample size determination. The total population of patients on follow-up during the study period were 744 and the calculated sample size was 225 subjects. The subjects were chosen by using systematic random sampling method.

#### Data collection, quality control and analysis

Data was collected through review of patients’ medical charts and patients interview by using data collection formats. The collected data was checked daily for completeness before it was entered into Epi Info version 7 and analyzed using the Statistical Package for the Social Sciences (SPSS) version 21. Categorical and continuous variables were presented using summary measures and tables. Multivariate logistic regression was used to estimate the strength of the association between independent variables and BP control. P-value < 0.05 was considered as statistically significant.

#### Consideration and procedures

Blood pressure was measured using a pre-tested mercury sphygmomanometer (adult size) and a stethoscope. A patient’s BP was taken while the patient was in a sitting position, from the right arm after the patient rested for at least 5 min before measurement. Two measurements of BP on a single visit were taken minimum 4 min apart, and the averages of the two readings were used to determine the BP level. Weight and height were measured with patients standing without shoes and wearing light clothing. Waist circumference was measured by using a flexible tape meter just above the iliac crest.

#### Operational definitions

*Hypertension* SBP ≥ 140 mmHg and/or DBP ≥ 90 mmHg or current treatment for hypertension with antihypertensive medication [6].

*Controlled hypertension* BP < 150/90 mmHg in hypertensive patients aged 60 or older, or BP < 140/90 mmHg in hypertensive patients aged less than 60 years and all ages of hypertensive patients with diabetes mellitus or nondiabetic chronic kidney disease [13].

*Treatment modification* titration or decrease in dose, addition of drug, discontinuation of a drug or drug replacement of a drug.

*Physically active* If an individual performs regular physical activities for > 150 min/week.

### Results

#### Socio-demographic characteristics of patients

Out of a total 225 patients enrolled in the study, more than half (55.6%) were females. Most of the patients were older than 60 years (40.4%) and urban residents (98.2%). Mean age of the patients was 55.2 ± 12.3 years (range 29–83) (Table 1).

**Table 1:** Socio-demographic characteristics of hypertensive patients on treatment at Zewditu Memorial Hospital

Characteristics	N	%
Sex		
Female	125	55.6
Male	100	44.4
Age (years)		
29–39	22	9.8
40–49	40	17.8
50–59	72	32
≥ 60	91	40.4
Residence		
Rural	4	1.8
Urban	221	98.2
Marital status		
Single	18	8
Married	159	70.7
Divorced	8	3.6
Widowed	40	17.8
Education level		
Cannot read and write	25	11.1
Primary level	68	30.2
Secondary level	70	31.1
Tertiary level	62	27.6
Occupation		
Government employed	59	26.2
Private employed	43	19.1
Housewife	46	20.4
Non-employed	17	7.6
Retired	53	23.6
Others*	7	3.1

Others* Merchant, farmer, bakery

Among the substances used by participants, salt, coffee and tea ranked the top three slots. Khat and alcohol did not seem to be commonly used by the participants (Additional file 1: Table S1).

A family history of hypertension was present in half (50.2%) of the patients, most of which (44%) were first degree relatives. Majority of the study subjects were above the normal BMI (54.7%) and waist circumstance (61.8%). The mean BMI was 25.88 ± 3.76 kg/m^2^ (range 16.18–41.91) and the mean waist circumstance was 98.42 ± 9.99 cms (range 69–127). Since starting drug therapy, patients were treated on average for 8.22 ± 7.07 years (range 0.5–39). Most study subjects were long-term hypertensives, with 55.1% of patients taking antihypertensive medications for more than 5 years. The median frequency of BP measurement was 17 times per year (Q_1_ = 4, Q_3_ = 52). About a third (29.3%) of the patients had some kind of associated comorbidity of which diabetes mellitus (DM) was the most frequent comorbidity (24%). Above half (53.3%) of patients were physically active (Additional file 2: Table S2).

#### Antihypertensive therapy

The mean number of antihypertensive drugs per patient was 2.25 ± 0.78 (range 1–4) and 16.9% of the patients were on monotherapy. With respect to the overall utilization of antihypertensive, ACEIs were the most commonly prescribed class followed by CCBs and BBs (Table 2).

**Table 2:** Distribution of antihypertensive drugs among patients receiving monotherapy and among total patients receiving antihypertensive drugs at Zewditu Memorial Hospital

Drugs	As monotherapy (N = 38) (%)	Total sample (N = 225) (%)
ACEIs	17 (44.7)	158 (70.2)
Enalapril	17 (44.7)	157 (69.8)
Lisinopril	0 (0)	1 (0.4)
CCBs	13 (34.2)	147 (65.3)
Nifedipine	12 (31.6)	141 (62.7)
Amlodipine	1 (2.6)	6 (2.7)
BBs	4 (10.5)	146 (64.9)
Atenolol	4 (10.5)	143 (63.6)
Metoprolol	0 (0)	1 (0.4)
Propranolol	0 (0)	2 (0.9)
Diuretics	1 (2.6)	51 (22.7)
Hydrochlorothiazide	1 (2.6)	41 (18.2)
Furosemide	0 (0)	10 (4.4)
Spirinolactone	0 (0)	1 (0.4)
α_2_-A		
Methyldopa	3 (7.9)	3 (1.3)
ARB		
Losartan	0 (0)	1 (0.4)

Total number of patients is greater than 225 because some were prescribed more than one class of drug

*ACEIs* angiotensin converting enzyme inhibitors, *ARB* angiotensin II receptor blocker, *BBs* beta blockers, *CCBs* calcium channel blockers, α_2_-A Alpha2-Agonist

The majority (83.1%) of the patients were on combination therapies. A greater number of patients (44.4%) were on two-drug therapy and about one third (35.1%) of patients were on three-drug therapy. The most commonly prescribed two-drug class combinations were ACEI + BB, CCB + ACEI and CCB + BB. Among those patients on three-drug combinations, the majority were on CCB + ACEI + BB (Table 3).

**Table 3:** Distribution of antihypertensive drug combinations among hypertensive patients at Zewditu Memorial Hospital

Combination of drugs	N = 225	%
Two drugs combination	100	44.4
ACEI + BB	32	14.2
Enala + ateno	32	14.2
CCB + ACEI	28	12.4
Nife + enala	25	11.1
Enala + amlo	2	0.9
Amlo + lisino	1	0.4
CCB + BB	25	11.1
Nife + ateno	21	9.3
Ateno + amilo	2	0.9
Nife + prop	2	0.9
CCB + diuretic	6	2.7
Nife + HCT	5	2.2
Nife + furo	1	0.4
ACEI + diuretic	5	2.2
Enala + HCT	5	2.2
BB + diuretic	4	1.8
Ateno + HCT	3	1.3
Furo + meto	1	0.4
Three drugs combination	79	35.1
CCB + ACEI + BB	53	23.6
Nife + enala + ateno	53	23.6
ACEI + BB + diuretic	10	4.4
Enala + ateno + HCT	8	3.6
Enala + ateno + furo	2	0.9
CCB + BB + diuretic	8	3.6
Nife + ateno + HCT	6	2.7
Nife + ateno + furo	2	0.9
CCB + ACEI + diuretic	6	2.7
Nife + enala + HCT	6	2.7
BB + diuretic + diuretic	1	0.4
Ateno + HCT + furo	1	0.4
CCB + BB + ARB	1	0.4
Nife + ateno + losa	1	0.4
Four drugs combination	8	3.6
CCB + ACEI + BB + diuretic	6	2.7
Nife + enala + ateno + HCT	5	2.2
Nife + enala + ateno + furo	1	0.4
ACEI + BB + diuretic + diuretic	1	0.4
Enala + ateno + HCT + furo	1	0.4
CCB + BB + diuretic + diuretic	1	0.4
Nife + ateno + furo + spir	1	0.4

*ACEI* angiotensin converting enzyme inhibitor, *ARB* angiotensin II receptor blocker, *BB* beta blocker, *CCB* calcium channel blocker, *HCT* hydrochlorothiazide, *Amlo* amlodipine, *Ateno* atenolol, *Enala* Enalapril, *Furo* Furosemide, *Lisino* Lisinopril, *Losa* losartan, *Nife* nifedipine, *Prop* propranolol, *Meto* Metoprolol, *Spir* spirinolactone

Drug treatment was modified for about 22% of patients on a single visit to the clinic. The most frequent types of treatment modification were dose titration (6.7%) and addition of a drug (5.3%). Treatments were most often modified because of increased BP (11.6%), followed by a decrease in BP (3.6%) and medication side effect (2.2%) (Additional file 3: Table S3).

#### Hypertension control

The mean SBP and DBP were 142.36 ± 17.37 mmHg (range 70–200) and 87.08 ± 9.58 mmHg (range 60–120), respectively. SBP was controlled in 42.7% of patients, and DBP was below 90 mm Hg in 40% patients. Both SBP and DBP were controlled in 26.2% of all patients (Additional file 4: Figure S1).

#### Determinants of hypertension control

In the multivariate logistic regression model, only two variables were shown to have an association with BP control among the investigated variables. The likelihood of better control increased by twofold in patients with 60 years of age and above (AOR 2.58, CI 1.27–5.24) compared to those younger than 60 years of age. By contrast, treatment modification (AOR 0.21, CI 0.07–0.65) was negatively associated with hypertension control (Additional file 5: Table S4).

### Discussion

The major finding of this study was that only a quarter of the hypertensive patients on antihypertensive treatment had controlled BP and ACEIs were the most commonly prescribed class of antihypertensive drugs followed by CCBs and BBs. Other important findings were that BP control was associated with age and treatment modification.

In the present study, the prevalence of controlled hypertension among hypertensive patients on therapy at Zewditu Memorial Hospital was low 59 (26.2%). This is in agreement with studies conducted in Ethiopia (25.6%) [11] and Cameroon (24.6%) [14]. On the other hand, studies conducted in Australia [15], Jordan [16] and USA [17] reported higher BP control. In this study, 42.7% and 40% of the participants reached target SBP and DBP, respectively. The proportion achieved the target SBP and DBP were lower than those reported in the studies conducted in Australia [15] and USA [17, 18]. This discrepancy might be due to race [19, 20], low socioeconomic status, poor adherence to standard guidelines, inappropriate choice of antihypertensive drugs by clinicians [6], poor adherence to treatment by hypertensive patients, physician inertia, deficiencies of healthcare systems in their approach to chronic diseases [21], lack of drug availability at health facility, unaffordability of drugs by patients, and less aggressive treatment [22].

In the present study, majority of patients who were on antihypertensive treatment (60%) were 60 years of age and younger and this investigation was comparable to the study conducted by Woldu et al. [23]. Patients 60 years of age and older were 2.5 times (AOR 2.58, CI 1.27–5.24) more likely to have their hypertension controlled. In agreement with the present study, Wang et al. [24] reported that old age was associated with higher control of hypertension. In Wang et al. [24] study, elders had more awareness and medication treatment. In contrast, other studies reported lower BP control rates among those with older age [17, 25, 26]. As comorbidities were more common among elders, there could be more frequent monitoring, earlier detection and treatment, and more aggressive treatment which may have contributed to good BP control in elderly patients. The other reason may be that health professionals could be more concerned in counseling and ordering appropriate management for elders. As a result, they become adherent to the ordered medications.

Among hypertensive patients in our study, 29.3% had written evidence of comorbidity and DM was the most frequent (24%) comorbid condition, which was lower than results of other studies [16, 22, 23]. This might be due to rare screening or under detection for comorbidities as comorbidities are usually silent.

In the present study, ACEIs, CCBs, and BBs were the common antihypertensive drugs prescribed either in monotherapy or combination or both. The prescription rate of diuretics was very low both as monotherapy (2.6%) as well as in the total sample (22.7%). This study is in contrary to other studies in Ethiopia [27], South Africa [20, 28] and USA [29], where diuretics were the major prescribed class followed by ACEIs, CCBs, and BBs. In this study low rate use of diuretics might be due to prevalence of comorbid conditions (i.e. DM). With regards to rate of diuretics utilization, this result might be comparable to Woldu et al. [23] study, where diuretics were reported to be the third ranking prescribed antihypertensives. This study further demonstrated that among the dual antihypertensive therapy the combination of ‘ACEI + BB’ was the most commonly prescribed regimen, while triple and four drug combination therapy were ‘CCB + ACEI + BB’ and ‘CCB + ACEI + BB + Diuretics’, respectively. This finding is quite different from other studies in Ethiopia [23], India [30] and Ivory Coast [31]. The prescription patterns of antihypertensives vary from one country to another. The possible reasons to explain this disparity included reimbursement policies, opinion leaders with conflicts of interests, traditions, domestic pharmaceutical production, and clinical practice guidelines [32]. Most patients (83.1%) received combination therapy. The higher prescription rate of combination therapy might be due to the hospital based patients presented with the severe and more complicated hypertension since they are referred from the lower level health facilities.

Drug treatment was modified for 22.2% of patients. Dose titration and the addition of a drug were the leading types of treatment modifications. This is because most of the present study participants had uncontrolled BP. In this study, in agreement with Andrade et al. [25] treatment modification (OR 0.21, CI 0.07–0.65) was negatively associated with hypertension control.

### Conclusion

BP control to target goal was low and achieved only in one-fourth of patients on antihypertensive therapy. Age 60 years and above was found to be a good determinant of BP control, while treatment modification was identified as a predictor of poor BP control. ACEIs were the most commonly prescribed medication followed by CCBs and BBs. Majority of the prescriptions were combination drug therapy. Dose titration was the most frequent type of treatment modification and an increase in BP or uncontrolled BP was the most frequent reason for treatment modification.

## Limitations

Since this study was conducted in one hospital, it is difficult to generalize the findings to the total hypertensive patients in the country. In addition, because of the cross-sectional nature of the study design, there was no assessment of whether the present therapy was the initial one or whether it replaced or amended the original one.

## Additional files

**Additional file 1: Table S1.** Frequency distribution of substances use among hypertensive patients on treatment at Zewditu Memorial Hospital.

**Additional file 2: Table S2.** Frequency distribution of anthropometrics and clinical characteristics among hypertensive patients on treatment at Zewditu Memorial Hospital.

**Additional file 3: Table S3.** Treatment modification among hypertensive patients at Zewditu Memorial Hospital.

**Additional file 4: Figure S1.** Blood pressure at goals among hypertensive patients at Zewditu Memorial Hospital.

**Additional file 5: Table S4.** Logistic regression analysis for the association between selected variables and blood pressure control among hypertensive patients at Zewditu Memorial Hospital.

## Abbreviations

ACEIs: angiotensin converting enzyme inhibitors
AHA: American Heart Association
ARB: angiotensin II receptor blocker
BBs: beta blockers
BP: blood pressure
CCBs: calcium channel blockers
CHD: coronary heart disease
CKD: chronic kidney disease
DM: diabetes mellitus
FDRE/MOH: Federal Democratic Republic of Ethiopia/Ministry of Health
HF: heart failure
JNC: Joint National Committee
NCDs: non-communicable diseases
SPSS: Statistical Package for the Social Sciences
α_2_-A: Alpha2-Agonist

## References

[1] WHO (2014). World health statistics 2014.

[2] WHO (2014). Global status report on noncommunicable diseases 2014.

[3] Lopez AD, Mathers CD, Ezzati M, Jamison DT, Murray CJL (2006). Global burden of disease and risk factors.

[4] FDRE/MOH. Health and health related indicators for year 2005/2006 in Ethiopia; 2006.

[5] Longo DL, Kasper DL, Jameson JL, Fauci AS, Hauser SL, Loscalzo J (2012). Harrison’s principles of internal medicine.

[6] Chobanian AV, Bakris GL, Black HR, Cushman WC, Green LA, Izzo JL (2003). The seventh report of the Joint National Committee on Prevention, Detection, Evaluation, and Treatment of High Blood Pressure: the JNC 7 Report. JAMA.

[7] Bissa S, Mossie A, Gobena T (2014). Prevalence of hypertension and its association with substance use among adults living in Jimma Town, South West Ethiopia. WJMMS..

[8] Gudina EK, Bonsa F, Hajito KW (2014). Prevalence of hypertension and associated factors in Bedele town, southwest Ethiopia. Ethiopa J Health Sci..

[9] Mengistu MD (2014). Pattern of blood pressure distribution and prevalence of hypertension and prehypertension among adults in Northern Ethiopia: disclosing the hidden burden. BMC Cardiovasc Disord..

[10] Nshisso LD, Reese A, Gelaye B, Lemma S, Berhane Y, Williams MA (2012). Prevalence of hypertension and diabetes among Ethiopian adults. Diabetes Metab Syndr..

[11] Tesfaye F, Byass P, Wall S (2009). Population based prevalence of high blood pressure among adults in Addis Ababa: uncovering a silent epidemic. BMC Cardiovasc Disord..

[12] FDRE/MOH. Health sector development program IV 2010/11—2014/15; 2010.

[13] James PA, Oparil S, Carter BL, Cushman WC, Dennison-Himmelfarb C, Handler J (2013). 2014 evidence-based guideline for the management of high blood pressure in adults report from the panel members appointed to the Eighth Joint National Committee (JNC 8). JAMA.

[14] Dzudie A, Kengne AP, Muna WFT, Ba H, Menanga A, Kouam CK (2012). Prevalence, awareness, treatment and control of hypertension in a self-selected sub-saharan African urban population: a cross sectional study. BMJ Open.

[15] Chowdhury EK, Owen A, Krum H, Wing LMH, Ryan P, Nelson MR (2013). Barriers to achieving blood pressure treatment targets in elderly hypertensive individuals. J Hum Hypertens.

[16] Bulatova NR, Yousef A, AbuRuz SD, Farha RA (2013). Hypertension management and factors associated with blood pressure control in jordanian patients attending cardiology clinic. Trop J Pharm Res..

[17] Ornstein SM, Nietert PJ, Dickerson LM (2004). Hypertension management and control in primary care: a study of 20 practices in 14 states. Pharmacotherapy..

[18] Singer GM, Izhar M, Black HR (2002). Goal-oriented hypertension management: translating clinical trials to practice. Hypertension.

[19] Fiscella K, Holt K (2008). Racial disparity in hypertension control: tallying the death toll. Ann Fam Med..

[20] Onwukwe SC, Omole OB (2012). Drug therapy, lifestyle modification and blood pressure control in a primary care facility, south of Johannesburg, South Africa: an audit of hypertension management. S Afr Fam Pract..

[21] Mancia G, Fagard R, Narkiewicz K, Redo´n J, Zanchetti A, Bo¨hm M (2013). ESH/ESC Guidelines for the management of arterial hypertension: the Task Force for the management of arterial hypertension of the European Society of Hypertension (ESH) and of the European Society of Cardiology (ESC). J Hypertens.

[22] Goverwa TP, Masuka N, Tshimanga M, Gombe NT, Takundwa L, Bangure D (2014). Uncontrolled hypertension among hypertensive patients on treatment in Lupane District, Zimbabwe, 2012. BMC Res Notes..

[23] Woldu MA, Shiferaw DF, Lenjisa JL, Tegegne GT, Tesafye G, Dinsa H (2014). Antihypertensive medications pattern and their effect in blood pressure control in patients attending Bishoftu general hospital ambulatory ward, Debrezeit (Bishoftu), Ethiopia. World J Pharm Sci..

[24] Wang H, Zhang X, Zhang J, He Q, Hu R (2013). Factors associated with prevalence, awareness, treatment and control of hypertension among adults in southern china: a community-based, cross-sectional survey. PLoS ONE.

[25] Andrade SE, Gurwitz JH, Field TS, Kelleher M, Majumdar SR, Reed G (2004). Hypertension management: the care gap between clinical guidelines and clinical practice. Am J Manag Care..

[26] De Macedo ME, Lima MJ, Silva AO, Alcantara P, Ramalhinho V, Carmona J (2007). Prevalence, awareness, treatment and control of hypertension in Portugal: the PAP study. Rev Port Cardiol.

[27] Alemkere G, Teshale C, Ergetie Z, Berhane A (2012). Patient related characteristics on the choice of antihypertensive medication in the elderly in Jimma University Specialized Hospital, Jimma, Oromia region, South West Ethiopia. Int J Res in Pharmacol Pharmacother.

[28] Maepe LM, Outhoff K (2011). Hypertension in goldminers. S Afr Med J.

[29] Asch SM, McGlynn EA, Hiatt L, Adams J, Hicks J, DeCristofaro A (2005). Quality of care for hypertension in the United States. BMC Cardiovasc Disord..

[30] Cidda M, Mateti UV, Batchu MK, Martha S (2014). Study of prescribing patterns of antihypertensives in South Indian population. Int J Basic Clin Pharmacol..

[31] Kramoh KE, Ak´e-Traboulsi E, Konin C, N’goran Y, Coulibaly I, Adoubi A (2012). Management of hypertension in the elderly patient at abidjan cardiology institute (Ivory Coast). Int J Hypertens.

[32] Fretheim A, Oxman AD (2005). International variation in prescribing antihypertensive drugs: its extent and possible explanations. BMC Health Serv Res..

