# Supplementary material for: Assessment of hypertension control and factors associated with the control among hypertensive patients attending at Zewditu Memorial Hospital: a cross sectional study
Source: BMC Res Notes. 2019 Mar 18;12:152. doi: 10.1186/s13104-019-4173-8 (PMC6423777; doi:10.1186/s13104-019-4173-8)
Supplement: Supplementary file 1 — Additional file 1: Table S1. Frequency distribution of substances use among hypertensive patients on treatment at Zewditu Memorial Hospital. [file 13104_2019_4173_MOESM1_ESM.docx]

Table S1: Frequency distribution of substances use among hypertensive patients on treatment at Zewditu Memorial Hospital.

| **Characteristics** | **N** | **%** |
| --- | --- | --- |
| **Alcohol use/day**  None  <1 drink  1-2 drinks | 208  8  9 | 92.4  3.6  4 |
| **Khat chewing/week**  None  <250 g  250-500 g | 220  4  1 | 97.8  1.8  0.4 |
| **Salt use**  No  Yes | 119  106 | 52.9  47.1 |
| **Coffee use/day**  None  1 cup  2 cups  ≥3 cups | 97  87  22  19 | 43.1  38.7  9.8  8.4 |
| **Tea use/day**  None  1 cup  2 cups  ≥3 cups | 129  63  24  9 | 57.3  28.0  10.7  4.0 |
