# Supplementary material for: Assessment of hypertension control and factors associated with the control among hypertensive patients attending at Zewditu Memorial Hospital: a cross sectional study
Source: BMC Res Notes. 2019 Mar 18;12:152. doi: 10.1186/s13104-019-4173-8 (PMC6423777; doi:10.1186/s13104-019-4173-8)
Supplement: Supplementary file 2 — Additional file 2: Table S2. Frequency distribution of anthropometrics and clinical characteristics among hypertensive patients on treatment at Zewditu Memorial Hospital. [file 13104_2019_4173_MOESM2_ESM.docx]

Table S2: Frequency distribution of substances use among hypertensive patients on treatment at Zewditu Memorial Hospital.

| **Characteristics** | **N** | **%** |
| --- | --- | --- |
| **Family history of hypertension**  First degree relatives  Second degree relatives | 113  98  16 | 50.2  43.6  7.1 |
| **Body mass index (BMI)**  <25 kg/m^2^  25-29.9 kg/m^2^  ≥30 kg/m^2^ | 102  98  25 | 45.3  43.6  11.1 |
| **Waist circumstance**  Normal WC  High WC (F > 88 cms & M > 102 ms**)** | 86  139 | 38.2  61.8 |
| **How often BP measure Per year**  4-12  13-52  >52 | 106  78  41 | 47.1  34.7  18.2 |
| **Duration of drug treatment**  0.5-2 yrs  3-5 yrs  6-8 yrs  9-11 yrs  >=12 yrs | 46  55  38  39  47 | 20.4  24.4  16.9  17.3  20.9 |
| **Comorbidity**  Diabetes mellitus  Cerebrovascular diseases  Hyperlipidimia  Chronic kidney disease  Heart failure  Coronary artery diseases | 66  54  10  8  2  2  1 | 29.3  24  4.4  3.6  0.9  0.9  0.4 |
| **Number of comorbidity**  0  1  ≥2 | 159  56  10 | 70.7  24.9  4.4 |
| **Physical activity**  Physically inactive  Physically active | 105  120 | 46.7  53.3 |

Where, BP, Blood pressure; WC, waist circumstance; cms, centimeters; F, female; M, male.
