# Supplementary material for: Assessment of hypertension control and factors associated with the control among hypertensive patients attending at Zewditu Memorial Hospital: a cross sectional study
Source: BMC Res Notes. 2019 Mar 18;12:152. doi: 10.1186/s13104-019-4173-8 (PMC6423777; doi:10.1186/s13104-019-4173-8)
Supplement: Supplementary file 3 — Additional file 3: Table S3. Treatment modification among hypertensive patients at Zewditu Memorial Hospital. [file 13104_2019_4173_MOESM3_ESM.docx]

Table S3: Treatment modification among hypertensive patients at Zewditu Memorial Hospital.

| **Characteristics** | **N** | **%** |
| --- | --- | --- |
| **Types**  Dose titration  Addition of drugs  Drug replacement  Dose decrease  Discontinuation of drugs | 15  12  9  9  5 | 6.7  5.3  4  4  2.2 |
| **Reasons**  Increase in BP  Decrease in BP  Medication side effect  Unknown reasons | 26  8  5  4 | 11.6  3.6  2.2 |
