# Supplementary material for: Assessment of hypertension control and factors associated with the control among hypertensive patients attending at Zewditu Memorial Hospital: a cross sectional study
Source: BMC Res Notes. 2019 Mar 18;12:152. doi: 10.1186/s13104-019-4173-8 (PMC6423777; doi:10.1186/s13104-019-4173-8)
Supplement: Supplementary file 4 — Additional file 4: Figure S1. Blood pressure at goals among hypertensive patients at Zewditu Memorial Hospital. [file 13104_2019_4173_MOESM4_ESM.docx]

**Figure** S1: Blood pressure at goals among hypertensive patients at Zewditu Memorial Hospital.
