# Supplementary material for: Assessment of hypertension control and factors associated with the control among hypertensive patients attending at Zewditu Memorial Hospital: a cross sectional study
Source: BMC Res Notes. 2019 Mar 18;12:152. doi: 10.1186/s13104-019-4173-8 (PMC6423777; doi:10.1186/s13104-019-4173-8)
Supplement: Supplementary file 5 — Additional file 5: Table S4. Logistic regression analysis for the association between selected variables and blood pressure control among hypertensive patients at Zewditu Memorial Hospital. [file 13104_2019_4173_MOESM5_ESM.docx]

Table S4: Logistic regression analysis for the association between selected variables and blood pressure control among hypertensive patients at Zewditu Memorial Hospital.

| Characteristics | BP control  No (%) Yes (%) | | COR (95% CI) | AOR (95% CI) |
| --- | --- | --- | --- | --- |
| Sex  Female  Male | 92  74 | 33  26 | 1.00  0.98(0.54-1.78) | 1.00  0.69(0.34-1.40) |
| Age (yrs)  <60  ≥60 | 109  57 | 25  34 | 1.00  2.60(1.42-4.78)* | 1.00  2.58(1.27-5.24)* |
| Monthly income  <600  600-1200  >1200 | 32  31  103 | 19  10  30 | 1.00  0.54(0.22-1.35)  0.49(0.24-0.99)* | 1.00  0.64(0.23-1.73)  0.69(0.31-1.51) |
| Family history of hypertension  No  Yes | 79  87 | 33  26 | 1.00  0.72(0.39-1.30) | 1.00  0.71(0.36-1.38) |
| Body mass index  <25  25-29.9  ≥30 | 71  73  22 | 31  25  3 | 1.00  0.78(0.42-1.46)  0.31(0.09-1.12) | 1.00  0.84(0.42-1.66)  0.32(0.08-1.28) |
| Comorbidity  No  Yes | 120  46 | 39  20 | 1.00  1.34(0.71-2.53) | 1.00  1.24(0.62-2.47) |
| Treatment modification  No  Yes | 127  39 | 55  4 | 1.00  0.24(0.08-0.695)* | 1.00  0.21(0.07-0.65)* |
| Number of drugs  Monotherapy  Polytherapy | 24  142 | 14  45 | 1.00  0.54(0.26-1.14) | 1.00  0.59(0.17-2.07) |
| CCBs  No  Yes | 58  108 | 20  39 | 1.00  1.05(0.56-1.96) | 1.00  1.19(0.49-2.85) |
| ACEIs  No  Yes | 45  121 | 22  37 | 1.00  0.63(0.33-1.17) | 1.00  0.65(0.28-1.54) |
| BBs  No  Yes | 54  112 | 25  34 | 1.00  0.66(0.36-1.21) | 1.00  0.95(0.40-2.27) |
| Diuretics  No  Yes | 131  35 | 45  14 | 1.00  1.16(0.58-2.36) | 1.00  1.77(0.71-4.40) |

Where, ACEI, Angiotensin Converting Enzyme Inhibitor; BB, Beta Blocker; CCB= Calcium Channel Blocker
